# Recent Progress on Physiologically Based Pharmacokinetic (PBPK) Model: A Review Based on Bibliometrics

**DOI:** 10.3390/toxics12060433

**Published:** 2024-06-14

**Authors:** He Huang, Wenjing Zhao, Ning Qin, Xiaoli Duan

**Affiliations:** School of Energy and Environmental Engineering, University of Science and Technology Beijing, Beijing 100083, China; huanghe19982023@163.com (H.H.); zhaowj0326@163.com (W.Z.)

**Keywords:** PBPK model, bibliometric, CiteSpace, species-specific parameter, risk assessment

## Abstract

Physiologically based pharmacokinetic/toxicokinetic (PBPK/PBTK) models are designed to elucidate the mechanism of chemical compound action in organisms based on the physiological, biochemical, anatomical, and thermodynamic properties of organisms. After nearly a century of research and practice, good results have been achieved in the fields of medicine, environmental science, and ecology. However, there is currently a lack of a more systematic review of progress in the main research directions of PBPK models, especially a more comprehensive understanding of the application in aquatic environmental research. In this review, a total of 3974 articles related to PBPK models from 1996 to 24 March 2024 were collected. Then, the main research areas of the PBPK model were categorized based on the keyword co-occurrence maps and cluster maps obtained by CiteSpace. The results showed that research related to medicine is the main application area of PBPK. Four major research directions included in the medical field were “drug assessment”, “cross-species prediction”, “drug–drug interactions”, and “pediatrics and pregnancy drug development”, in which “drug assessment” accounted for 55% of the total publication volume. In addition, bibliometric analyses indicated a rapid growth trend in the application in the field of environmental research, especially in predicting the residual levels in organisms and revealing the relationship between internal and external exposure. Despite facing the limitation of insufficient species-specific parameters, the PBPK model is still an effective tool for improving the understanding of chemical–biological effectiveness and will provide a theoretical basis for accurately assessing potential risks to ecosystems and human health. The combination with the quantitative structure–activity relationship model, Bayesian method, and machine learning technology are potential solutions to the previous research gaps.

## 1. Introduction

Physiologically based pharmacokinetic/toxicokinetic (PBPK/PBTK) models are established on the basis of physiological and biochemical properties of organisms, anatomical properties of organisms, and thermodynamic properties of drugs [1]. The target tissues and organs to be studied are treated as a single compartment, and the modules are connected to each other by the blood circulation system [2]. Each module realizes the drug transport in the body according to the principle of material balance under the control of cardiac output, blood flow rate, drug properties, and tissue–plasma partition coefficient. The pharmacokinetic data are processed by computer means, and the final model can be used for calculating the concentration of the drug in the various organs of the organism. Based on the model, the behaviors of drugs or chemicals in the organism can be quantitatively characterized, and intraspecies extrapolation and interspecies extrapolation can also be carried out for tissue residual-level prediction [3].

The concept of PBPK modeling was first introduced by Teorell in 1937, when he introduced mathematical equations to describe changes in drug concentrations in blood and body tissues over time [4]. In 1960, Bellman modeled the relationship between capillaries, cells, and cellular interstitial spaces [5]. It was not until 1973 that Dedrick introduced the concept of scaling up, which opened animal–human extrapolation [6]. Due to the limitations of computer technology at that time, the progress of the PBPK model was slow. Nowadays, with advancements in computer technology, the structure of the PBPK model ranges from a simple minimal setting, consisting of only a few basic compartments, to a whole-body PBPK model, in which all major organs of the body are represented by compartments linked through blood [7].

Nowadays, the ability of PBPK models to integrate information from various sources has greatly increased, and the PBPK method has been used for various extrapolation linkages, such as inter-route extrapolation [8,9], inter-dose extrapolation [10], and inter-species extrapolation [11,12]. Previously, allometric scaling was the most common method for inter-species extrapolation [13,14]. However, in a retrospective study comparing the accuracy of the allometric scaling method and the PBPK method, the performance of the PBPK method was better than the allometric scaling method in predicting human plasma concentration–time curves from animal data [15]. Another important application of the PBPK model is to quantify exposure to remote and inaccessible regions, such as the brain or tumor [16,17]. As for the population targeted by the model, it was initially more applicable to normal people or adult patients, and now it is also applicable to special populations such as fetuses [18], newborns [19], and pregnant women [20]. Although most PBPK modeling-related applications involve rodents and humans, more species such as sheep [21], ponies [22], fish [23], and oysters [24], among others, are now also used in physiological modeling exercises.

Besides the application in medical research. Much attention has been paid to the PBPK application in environmental research. The bioavailability of a contaminant is defined as its utilization by organisms after uptake and its potential harm to the organism [25]. Characterizing the bioavailability of pollutants can accurately assess the risks of pollutants. However, current risk assessment is usually based on the external exposure concentration, which will inevitably result in biological evaluation bias due to the neglect of bioavailability. Due to the ability of PBPK to quantitatively establish the relationship between external and internal exposure levels, models are considered an important method for assessing bioavailability in environmental research. In addition, parameters representing the physiological properties of organisms and thermodynamic properties of drugs were applied in the establishment of the model. Therefore, it is widely believed that the PBPK method can better characterize the distribution of substances in the body of the animal. However, in the existing studies, many relevant parameters for specific species are still missing, which makes the models less accurate in their predictions. There have been some reviews summarizing the progress related to PBPK, but existing reviews focus either on exploring modeling methods or on studying the metabolic kinetics of a certain type of pollutant [1,26]. Moreover, there is still a lack of comprehensive understanding of the research progress of the method. The aim of this research was to summarize the application in both the medical field and environmental research, review the development trends, and predict the future breakthrough directions of the PBPK model. We also focus on the application of the PBPK model in environmental field research.

## 2. Methods

### 2.1. PBPK Modeling

The PBPK model is based on the same mathematical framework as the classical Pharmacokinetic (PK) model. The compartments of a PBPK model correspond to different organs or tissues and incorporate the biological and physiological components of each organ or tissue. The data required for PBPK models include physiological parameters, drug-related parameters, and experimental design parameters. The target tissues and organs to be studied are treated as a single compartment, each compartment can construct a mathematical model (1), where *K* is the turnover rate of the compartment.

(1) $$\frac{dx}{dt}=-Kx\left(t\right)$$

Usually, compartments were used to represent the central tissues of the body including fat, bone, brain, intestine, heart, kidney, liver, lung, muscle, skin, and spleen [1]. The liver and kidney, as the main metabolic and excretory organs of the fish body, play an important role in the process of pollutant accumulation and elimination. These two organs are usually regarded as two separate compartments in the PBPK model [27,28]. The remaining tissues were allocated according to the rate of blood perfusion. Those with a high rate of blood perfusion had fully perfused tissue, while those with a low rate of blood perfusion had slowly perfused tissue. These tissue systems were regarded as compartments. The mass conservation differential description of various organizations in the model can be expressed as follows:

Liver:

(2) $$V_{l}\frac{dC_{l}}{dt}=Q_{l}\left(C_{a}-\frac{C_{l}}{P_{l}}\right)+Q_{r}\left(\frac{C_{r}}{P_{r}}-\frac{C_{l}}{P_{l}}\right)-V_{max}\left(\frac{\frac{C_{l}}{P_{l}}}{K_{m}+\frac{C_{l}}{P_{l}}}\right)$$

Kidney:

(3) $$V_{k}\frac{dC_{k}}{dt}=Q_{k}\left(C_{a}-\frac{C_{k}}{P_{k}}\right)-0.6Q_{p}\left(\frac{C_{p}}{P_{p}}-\frac{C_{k}}{P_{k}}\right)$$

Fully perfused tissue:

(4) $$V_{r}\frac{dC_{r}}{dt}=Q_{r}\left(C_{a}-\frac{C_{r}}{P_{r}}\right)$$

Slowly perfused tissue:

(5) $$V_{p}\frac{dC_{p}}{dt}=Q_{p}\left(C_{a}-\frac{C_{p}}{P_{p}}\right)$$

When modeling substances with long half-lives or persistent organic pollutants, the equation assumes a uniform distribution in the organization as [29]:

(6) $$\frac{dA_{t}}{dt}=Q_{t}\left(C_{a}-\frac{C_{t}}{P_{t:b}}\right)$$

In the formula, *A_t_* represents the amount of chemical in the compartment (μg), *C* represents the concentration of drugs in the compartment (μg L^−1^), *V* represents the volume of the organ (L), and *Q* represents the plasma flow rate in the organ (L h^−1^). Subscripts *a*, *p*, *k*, *r*, *l*, *v*, and *t* represent arterial blood, slowly perfused tissue, kidney, fully perfused tissue, liver venous blood, and the tissue, respectively. *V_max_* is the maximum metabolic rate constant (μg L^−1^ (kg bodyweight)^−1^). *K_m_* is the Michaelis constant (μg L^−1^). *P_t:b_* is the tissue: blood partition coefficient for the compartment. We should express the conservation of mass equation based on the different properties of matter.

### 2.2. Literature Search Strategy Bibliometrics Analysis

First, a systematic literature review was conducted based on the WOS database. PBPK and PBTK have the same meaning, so the keywords we searched for in our literature were “(((TS = (Physiology based pharmacokinetic model)) OR TS = (PBPK)) OR TS = (Physiology based toxicokinetic model)) OR TS = (PBTK)”. On this basis, progress in literature research in specific fields literature was screened again from all PBPK-related articles. Dissertations, duplicates, conferences, incomplete, or unavailability of original documents were excluded. The search spans an unlimited period. The retrieval date was 24 March 2024, and 3974 articles were obtained.

Bibliometric analyses were conducted by CiteSpace to study the research progress related to PBPK. CiteSpace is a citation visualization and analysis software that focuses on analyzing the potential knowledge embedded in scientific analyses and has evolved in the context of scientometrics and data visualization (https://citespace.podia.com/, accessed on 20 March 2024). CiteSpace software (6.3.R2, developed by Prof. Chaomei Chen) was used to import the filtered articles into CiteSpace 6.3.R1 for visual analysis. First, clustering analysis was conducted on the keywords to determine their frequency of occurrence. This can help determine the changes in research direction and focus in the field. Then, the visual analysis was carried out with the keywords of the country, and the time zone map of the country was drawn. The larger the circle, the more articles related to PBPK were published in that country. The richer the color, the earlier the country started related research. Finally, the number of documents issued by PBPK every year was analyzed to learn about the trends in documents issued.

## 3. Research Progress Based on Bibliometrics

### 3.1. Volume of Publications Analysis

**Figure 1:**
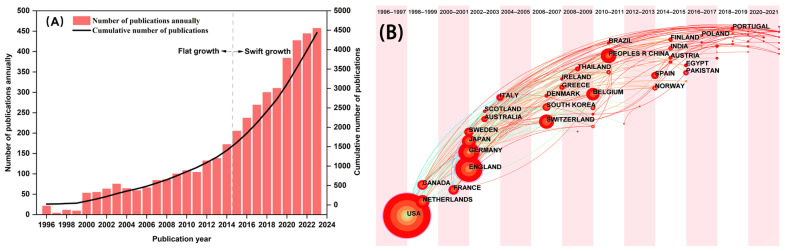
(**A**) Number of research papers on PBPK/PBTK. The dotted line represents changes in the rate of publication (**B**) The time zone view of co-occurrence network map of countries. Node size signifies the number of papers that originated from the country.

The results of the statistical analysis of the PBPK-related literature in the WOS databases are shown in Figure 1. It was found that the number of publications increased from 2 in 1996 to 457 in 2023. According to the statistical results of the first three months (188 articles), the number of publications in 2024 is likely to exceed that in 2023. It can also be seen from Figure 1A that the number of publications has a slower growth rate from 1996 to 2014. The number of papers increased rapidly after 2014. Especially in 2015, the number of papers published increased by 14.6% compared with the number in 2014. Increasing advanced computer science has made it possible for the calculation of complex mathematical equations for PBPK, which could be the potential reason that indirectly promoted PBPK research from 2014 to 2024. To date, the total number of publications is nearly 4000.

The data were input into CiteSpace to learn about the status of studies on the PBPK model from different countries. Generally, the major studies in this area were mainly conducted by developed countries, such as the USA, Netherlands, Canada, France, England, Germany, Japan, Sweden, Australia, Italy, Switzerland, Korea, Belgium, and Spain. Statistics by number of articles issued (Figure 1B), the USA leads the pack with a total of 1174 articles, followed by England with a publication number of 465, and Germany and China were the third and fourth place with publications of 260 and 170, respectively. The USA is also the first country to conduct research on PBPK. The first paper was published in 1996. Netherlands and Canada started their studies on PBPK in the years 1998–1999. As the only developing country in the top 15, China started the research on PBPK in 2010.

### 3.2. Keyword Co-Occurrence Clustering Analysis

**Figure 2:**
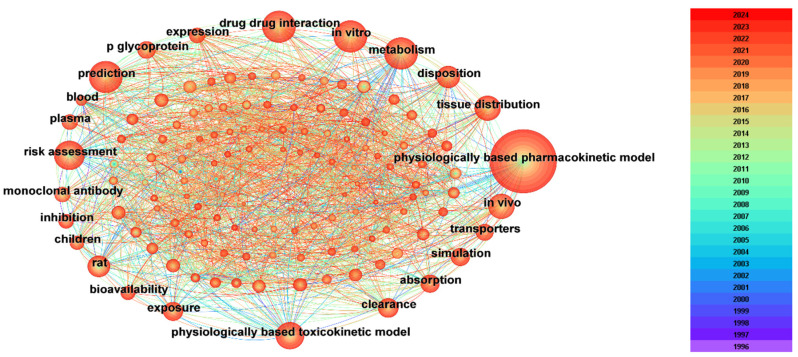
Map of co-occurring keywords. The circle represents the keyword cited in the PBPK research. The larger the diameter of the circle, the more frequently the keywords appear. The color represents the year of the publication. The richer the color of the circles and connecting lines, the longer the time span over which the keyword appears.

Keywords are usually considered as the summary of the research themes and contents of the literature. Therefore, keyword co-occurrence network analysis can reflect the development history and research hotspots of the relevant research fields, which can further help researchers quickly understand the dynamic evolution of each relevant knowledge unit in the field. In this study, articles related to the PBPK model screened from the WOS database were imported into CiteSpace and visualized and analyzed by keyword as node type to obtain the keyword co-occurrence network spectrum (Figure 2).

After removing less important keywords in the network view (Figure 2), it can be found that the most frequently occurring keywords were “metabolism” (391 times), “risk assessment” (345 times), “drug–drug interaction” (313 times), “prediction” (311 times), “in vitro” (309 times), “tissue distribution” (219 times), “in vivo” (211 times), “disposition” (200 times), “exposure” (168 times), etc. (Table 1). In addition, the centrality of the keywords was also applied to represent the importance of the keyword in the network. The centrality of a node is determined by the number of lines connecting to the node. Nodes with centrality greater than 0.1 are considered important nodes in a network. The keywords with co-occurrence network centrality greater than 0.1 included “risk assessment” (0.65), “metabolism” (0.37), “exposure” (0.16), “clearance” (0.13), and “prediction” (0.12) (Table 1). It can be found that some keywords including “prediction”, “exposure”, “risk assessment”, “special group”, “new drug development”, and “species extrapolation” clearly indicated the hotspots of PBPK research; the other keywords including “in vitro”, “in vivo”, “clearance”, “absorption” “disposition”, “metabolism”, “exposure”, and “partition coefficients” can be classified as the method of model construction and the important parameters that were needed. 

**Figure 3:**
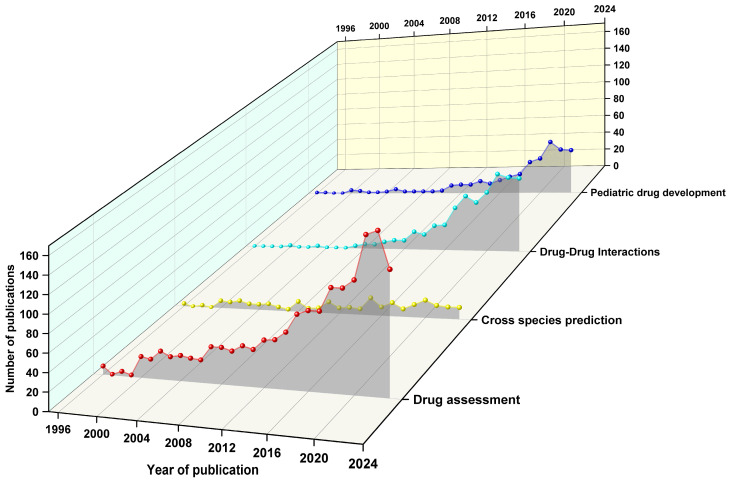
Number of publications in four research areas of the PBPK models.

As an important research target, the circle of “risk assessment” has both high occurrence frequency and a long research history. It can also be found that the circle of “risk assessment” has a close relationship with the circles of “exposure”, “rats”, and “tissue distribution”, which indicates that it is widely used in research focused on drug assessment and drug tests. The label “in vitro” has links with the circles of “rats”, “human”, and “prediction”. They are always used in the animal-to-human extrapolation of drugs or chemicals. The keyword “DDI” has associations with labels including “inhibition”, “pharmacokinetics”, and “metabolism”, which indicates another research hotspot of “drug–drug interactions”. Finally, the circle of “children” is close to “humans”, “in vitro”, and “exposure”, which indicates another application of PBPK, the research on pediatric drug development. Therefore, the existing articles were categorized into four main areas based on the frequency of keyword occurrences (Figure 3), including “drug assessment”, “cross-species prediction”, “drug–drug interactions (DDI)”, and “pediatric drug development” [3,26].

## 4. Application in Medical Field

### 4.1. Drug Assessment

The very earliest framework of the PBPK model was designed to describe the relationship between drug concentrations in blood and simulate the drug disposition. Therefore, high-occurrence keywords and their relationships with each other can be obtained through the cluster analysis of CiteSpace software. Keywords including “prediction”, “in vitro”, “in vivo”, “metabolism”, “tissue distribution”, and “absorption” were found to have a close relationship with drug assessment (Figure S1).

The number of articles related to drug evaluation is the greatest among the four areas, accounting for 55% of the total publications (Figure 3). There has been a high volume of publications in this area since 2003. The number of articles began to increase rapidly after the year of 2017. The fastest growth in this area was reported in 2021 with a rate of 39%. It can be concluded that “drug assessment” is the most concerned research area of PBPK research.

The fate of potential drug candidates is characterized in in vitro and preclinical in vivo systems by PBPK models (Table 2). The modeling of methylene chloride (DCM; dichloromethane) was the first quantitative use of the PBPK method in risk assessment, where a PBPK model was used to estimate the tissue dose of reactive metabolites [30]. Since the research on DCM, the PBPK approach has been used in the fate simulation of many different compounds to support dosimetry estimates in humans as part of the risk assessment process. Processes including absorption, distribution, metabolism, and excretion (ADME) of anticancer drugs [31], antibody drugs [32], and novel prodrugs have been reported by previous PBPK studies [33]. For example, Kim et al. provided valuable insights into the human health risk assessment of perfluorooctanesulfonic acid exposure [34]. Besides the application in drug assessment, PBPK models have been successfully applied in the research of the environmental influence of organic pollutants, including antibiotics, pesticides, and herbicides.

### 4.2. Cross Species Prediction

Cross-species prediction refers to the process of extrapolating in vivo experimental data to human in vivo physiology parameters through biological scaling factors [40]. After using keyword clustering analysis, “prediction”, “rat”, “binding”, “human”, “in vitro”, “in vivo”, and “blood” were found to have a close relationship with the research on cross-species prediction (Figure S2).

According to the statistical results of annual publication volume (Figure 3), the cross-species prediction of annual publication volume has been on a relatively stable trend. The lowest number of papers was in 2007, with only 2 articles published. The highest were in 2015 and 2020, with 18 articles published. Due to the possibility of obtaining pharmacokinetic data through interspecies extrapolation, research on cross-species prediction still plays an irreplaceable role in PBPK research.

Although the concept of PBPK emerged early, “top-down” remained the popular modeling strategy for pharmacokinetic models for a long time. The information for the model can only be obtained from a given pharmacokinetic and covariate data set. Therefore, the development of PBPK was limited by insufficient pharmacokinetic data in humans. With advancements in cross-species prediction, the “bottom-up” method was established by collecting pharmacokinetic data from animals to improve PBPK models. Therefore, the information from a priori physiological and pharmacological mechanisms can be used to improve the model [53]. Brinkmann developed PBTK models for zebrafish (Daniorerio) and roach (Rutilus rutilus) and combined them with existing models for rainbow trout (Onchorhynchus mykiss), lake trout (Salvelinus namaycush), and fathead minnow (Pimephales promelas). The resulting multi-species model framework allows extrapolation of the bioaccumulation potential of neutral organic compounds across species. The results predicted by the model are compared with the experimental data, and it is found that the prediction of most substances is accurate. Such models can therefore place particular emphasis on cross-species evaluations [54]. Loccisano established a PBPK model of perfluorinated alkyl acids (PFAA) in monkey tissues. The results of the monkey model are consistent with existing monkey PK data. Then, the monkey model was extrapolated to humans and successfully simulated data collected from residents of two communities who were exposed to PFOA through drinking water. The data for this model exhibit reasonable agreement with the available human serum PFOS data [40]. Cross-species extrapolation has also been applied in pediatric research. Jeremy et al. used pregnant sheep to establish a p-PBTK model to predict the health risks of bisphenol s (BPS) and bisphenol a (BPA) in the environment. These predictive simulations show fetal accumulation of both bisphenols over time. These models advance our understanding of bisphenolic compound toxicokinetics during pregnancy [55]. In cases of cross-species prediction, extrapolated data can be obtained by more than just modeling one animal. Yang et al. predicted the human pharmacokinetic spectra based on the physicochemical properties, pharmacokinetics, and tissue distribution data collected from mice and the pharmacokinetic spectra of hamsters [33] (Table 2).

### 4.3. Drug–Drug Interactions (DDIs)

Patients in clinical settings are often exposed to multiple medications to treat concurrent diseases or to effectively treat a single disease [56]. Co-administration of multiple drugs may increase the incidence of DDIs [57], which means a reduction in the effectiveness and safety of drugs and the possibility of potentially fatal adverse reactions [58]. Meanwhile, clinical trials are expensive and potentially risky, so the adoption of PBPK models provides a better choice for predicting human DDI in vitro (Table 2). The results of keyword cluster analysis indicated that “P glycoprotein”, “ketoconazole”, and “prediction” were closely linked to the topic (Figure S3).

After organizing articles in the field of DDI (Figure 3), we found that the annual publication volume from 2003 to 2010 was less than 10 articles. From 2004 to 2006, no related articles were published. After 2011, the growth rate of articles related to DDI began to accelerate, reaching a peak of 59% in 2015, with 10 more articles published than in 2014. It is worth noting that articles related to DDI have the highest publication volume in 2021, with a total of 84 articles. However, the trend of publication volume in the next two years began to decline.

PBPK studies on antineoplastic and immunomodulatory drugs were the most important topics of concern in this area, followed by studies on cardiovascular and anti-infective drugs [59]. Models for specific products such as herbal products, therapeutic protein drugs, and antibody–drug conjugates were also described [60]. Most PBPK research focused on simulating cytochrome P450 (CYP)-mediated DDIs [61], whereas some were used on transporter-mediated DDIs or a combination of CYP and transporter-mediated DDIs [62]. Multiple models have been established by previous research to study DDIs. All the models are usually classified into three main categories including simple static, mechanistic static, and mechanistic dynamic models. For simple static models, the quantification of DDI potentials is mainly based on a single constant inhibitor concentration and inhibition constants from in vitro data. Therefore, it represents the worst-case scenario where the size of the DDI may be overestimated [63]. For the mechanistic static model, the substrate drug is assumed to be metabolized not only in the liver but also in the intestines [64]. Nevertheless, the model is not capable of describing the complete dynamic characteristics of drug metabolism in humans [65]. PBPK models, which belong to mechanistic dynamic models, aim to explain all pharmacokinetics characteristics of a drug and describe time-variable concentrations of the substrate and inhibitor drug in different organs. Therefore, temporal profiles of inhibition procedures are defined, and the model has been shown to be more predictive than static models [66]. For example, the prediction of the DDI potential of AZD2066 as a perpetrator using the simple static model indicated that the occurrence of clinically significant DDIs is possible. However, the likelihood of DDIs occurring in vivo was low when the same in vitro data were analyzed using the PBPK model. The in vivo study indicated no or low risk for clinically significant DDIs, and this improved accuracy may be partially attributable to the ability of the PBPK model to use time-variable drug concentrations instead of a single inhibitor concentration [67].

### 4.4. Pediatrics and Pregnancy Drug Development

Pediatric drug development mainly aims at infants, children, and pregnant women who are more sensitive or prone to exposure (Figure S4). After keyword clustering of articles collected, it was found that “children”, “pregnant women”, “prediction”, “safety”, and “infants” appeared most frequently.

In the study of “pediatrics and prediction”, the annual number of articles published from 2003 to 2011 was less than 10, indicating a very slow attention. The rate of publication significantly accelerated since 2012, with a peak growth rate of 64% in 2019. In the past two years, there was a slight decrease in the number of articles published, but the publications on “pediatrics and prediction” remained at around 50 per year (Figure 3).

Compared with adults, infants, children, and pregnant women are sensitive to drugs and medicines. Considering the need for clinical studies in pediatric patients, the research and development of pediatric drugs has gradually become a new research area of clinical pharmacology. The application of PBPK in special populations provides not only a method to predict the exposure of the mother, placenta, and fetus to drugs through models, but also information to develop pediatric drugs based on adult physiological data. Duan et al. established an adult PBPK model for the renally excreted drugs linezolid and emtricitabine. PBPK models were then utilized to predict pharmacokinetics in pediatric patients for various age groups from the oldest to the youngest patients in a stepwise approach [68]. Biesdorf et al. established a PBPK model for ziprasidone and validated it in non-pregnant populations (healthy adults, elderly) and extended it to the pregnant state to evaluate the pharmacokinetic changes in ziprasidone throughout pregnancy [20]. Moreover, considering the difficulty of sampling for pediatrics and pregnancy, the PBPK model is also used for the risk assessment of pediatrics and pregnancy exposed to environmental toxic chemicals. PBPK models are now used to assess the risk of unintentional exposure to compounds in unborn and breastfed infants [69] and children [70,71], as well as the risk of neonatal exposure to environmental factors such as caffeine and theophylline [72] (Table 2).

## 5. Application of PBPK Model in Environmental Research

### 5.1. Simulating the Environmental Behaviors of Aquatic Pollutants

In recent years, more attention has been paid to the application of PBPK in environmental research, especially in aquatic ecosystems. Out of all the 3974 articles related to PBPK, a total of 145 were focused on the behavior of pollutants in aquatic animals. Water pollution has been one of the major environmental problems, especially for developing countries [73]. Heavy metals, trace elements, and various organic pollutants in aquatic environments are usually biologically accumulative, highly toxic, and difficult to degrade, which means great harm to both the ecological environment and human health [74]. Even worse, these pollutants vary widely in physicochemical properties and have different environmental behaviors. Therefore, PBPK is recommended as an effective method in pollutant behavior modeling, bioavailability estimation, and metabolism studies of pollutants.

**Figure 4:**
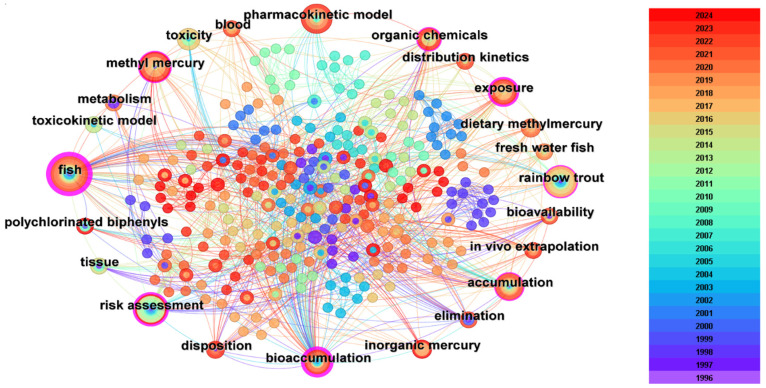
Co-occurrence network of keywords in the aquatic-related PBPK research.

According to the keyword co-occurrence map (Figure 4), the PBPK model is highly related to the keywords including blood, distribution kinetics, and bioaccumulation, which indicates that PBPK is highly applied in the field of pollutant ADME simulation and chemical tissue-level prediction. For example, Wintermyer et al. established the PBPK model and predicted the content of 2,3,7,8-tetrachlorodibenzo-p-dioxin (TCDD) in eastern United States marine oysters. The distribution and elimination concentration of dioxins in each tissue were further estimated, the results indicated the PBPK model successfully predicted the distribution and elimination concentration of TCDD in each tissue compartment of oyster. The model can also be used as a useful tool to predict the dynamics of other persistent organic pollutants. Some scholars have applied three dynamic models (biokinetic model: BK, physiologically based pharmacokinetic model: PBPK, and toxicokinetic–toxicodynamic model: TKTD) to understand the problem of metal ecotoxicology in aquatic systems. The results show that the PBPK model is more physiologically based, and it reveals the transport, transformation, and distribution of metals in organisms. The model provides guidelines for the further study of metal bioaccumulation and toxicity in aquatic organisms [75].

From Figure 4, it can be found that the model is also highly connected with keywords including metabolism, elimination, and bioavailability, which indicates that PBPK is also used to explore the biological metabolic process of pollutants. Liesbeth Weijs et al. tested the predictive ability of the model on the trend of polychlorinated biphenyl (PCB) concentration changes in porpoises in Beihai Bay from 1990 to 2002. This was the first study to compare the kinetics of several PCBs, evaluate their temporal trends, and attempt to reveal the metabolic pathways of marine mammalian species through the PBPK model. The utility of this biological monitoring has been greatly expanded, which can further deepen the understanding of the pharmacokinetics of chemicals of interest in these marine mammals and help explain the toxic effects revealed by their in vitro research results [76,77].

We can also find that keywords such as “freshwater fish” and “rainbow trout” were highly cited in related research, which means that studies on fish have received special attention. A total of 58 papers among all the 145 articles on aquatic environment focus on fish. Fish in natural water bodies are both a major “sink” for pollutants in the environment and a “source” of exposure to humans [78]. By checking the title of the papers, we can find that this research focuses on topics including behavior simulation of chemicals in fish, residual level prediction, and human risk assessment caused by polluted fish consumption. In order to better explore the transformation process of inorganic pollutants in fish bodies, radioisotopes were used as a tracer in the PBPK model by Zhang et al. to simulate the biological migration (absorption, distribution, elimination) and transformation of As (V) after aqueous exposure. The results indicated that the intestine and gills were the absorption, distribution, and elimination sites of As (V), while the carcass and head were the main storage sites. This is also the first time that modeling and simulation have been combined with biological transformation [79]. In addition, experts have developed and validated the PBTK-TD model of adult zebrafish exposure to Cd and Pb. The PBTK sub-model can accurately describe and predict the uptake, distribution, and disposition kinetics of Cd and Pb in zebrafish. Such models help us understand the fundamental processes regulating metal uptake and disposal in zebrafish and to quantitatively predict metal toxicity. More examples of the application of PBPK models on inorganic compounds are shown in Table 3. Compared with inorganic pollutants, more attention was paid to the metabolism of organic compounds in fish. Generally, the PBPK model is more effective than the other models in revealing the metabolic pattern and assessing the environmental risk of organic pollutant ontogeny and its metabolites. Stadnicka compared the goodness of prediction between the single-compartment model and the PBPK model by simulating the residual level of 39 organic chemicals in the tissues of two species of fish. As a result, the PBPK model outperformed the single-compartment model in simulating whole-body chemical concentrations with at least 88% of in vivo concentrations predicted to be within one order of magnitude [80]. Therefore, exploring the pollutant behavior of fish can not only assess the risk of pollutants in ecosystems, but also estimate the health risks that humans are exposed to through the consumption of fish.

### 5.2. Estimation of the Adverse Effects on Human Body

**Figure 5:**
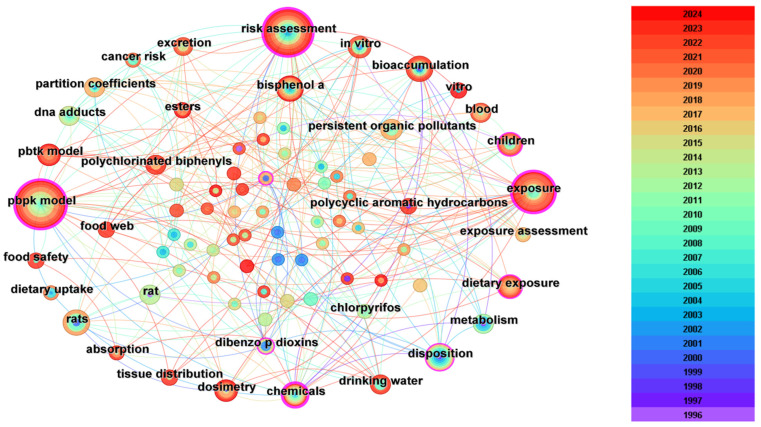
Co-occurrence network of keywords in the health-related PBPK research.

PBPK is often applied to estimate the adverse health effects on humans through different exposure pathways. Articles related to PBPK and health were searched and the retrieved literature was imported into CiteSpace to obtain the keyword co-occurrence map (Figure 5).

From Figure 5, it can be found that food exposure is the exposure route of greatest concern. Recent research has proved that food is highly related to internal exposure levels of POPs. The PBPK model showed that the estimated blood POP concentration increased significantly after food intake. For individuals with high internal burdens of POPs accumulated over many years, estimated blood levels were less affected by recent meal intake. In addition, the model estimated that the POP burden in the body is high even within a few years after the dietary shifts described above, and that contaminants accumulate 2–6 times faster than they decay after shifting to a lower-contaminant diet [85]. In addition, food types are also an important factor affecting POP exposure. Whales, seals, fish, and several species of marine life are part of the conventional diet of the Greenlander Inuit. A PBPK model was developed to estimate the fate of persistent organic pollutants (POPs) in the liver, blood, muscle, and adipose tissue of Greenlander Inuit following long-term exposure to a conventional Greenlandic diet. The model results showed a significant correlation between POP blood concentrations and calculated daily intake of POPs. Another study combined the PBPK model and the Weibull to assess the human health risk caused by consuming raw and cooked fish raised in groundwater arsenic-contaminated ponds in Taiwan. Simulation results showed that the health risk to humans caused by consumption of baked as-contaminated fish was <10^−6^ excess bladder cancer risk level for lifetime exposure. In contrast, contaminated fish cooked by frying resulted in significant health risks and a higher cumulative incidence ratio of liver cancer [86]. Based on these examples, we believe that it is highly necessary to use PBPK models as a tool for future human health exposure and impact assessments.

## 6. Research Gaps and Prospects

### 6.1. Research Gaps

PBPK has begun to receive attention and is widely used in medical and environmental fields. However, challenges still exist in PBPK building and application. The lack of parameters is the main problem affecting PBPK building and accuracy. The parameters needed for PBPK modeling can be classified into physiological parameters, drug-related parameters, and experimental design parameters. Physiological parameters include body weight, tissue volume, cardiac output, tissue perfusion rate, organ blood flow, etc. In addition, PBPK models are highly sensitive to the drug-related parameters including chemical-specific absorption, distribution, metabolism, and excretion parameters [82]. However, both types of important parameters necessary for PBPK can only be obtained experimentally, which is costly and time-consuming.

Although the problem has partly been resolved by using parameters from inter-species exploration, the parameters of different species differ greatly, so the parameters unique to the same species, so-called “species-specific” parameters, are important in PBPK modeling. Weijs found that some parameters from other species were proven to be inadequate for these models [77]. Studies also suggest that the application of PBPK models in the risk assessment of chemicals is limited by the lack of sophisticated parameters, especially species-specific parameters [87]. Therefore, the lack of species-specific parameters is still the major challenging issue in PBPK building.

### 6.2. Developing of the Modeling Strategies

Influenced by the lack of parameters, the modeling strategy of PBPK is changing. PBPK is a type of compartmentalized model. The compartments of a PBPK model correspond to different organs or tissues and incorporate the biological and physiological components of each organ or tissue. Compartmental models are traditionally developed using a ‘top-down’ approach where all of the information for the model comes from a given pharmacokinetic and in vivo data. Due to the application of published pharmacokinetics drug data as well as published physiological parameters of organs, “top-down” models have the advantages of completeness, adaptability, and clarity [88]. Meanwhile, insufficient in vivo data also limit the use of PBPK. Therefore, more and more in vitro data, along with the “bottom-up” strategy, are being used by the PBPK model. 

Compared with the “top-down” strategy, “bottom-up” approaches start from the constitutive parts by formulating the interactive behavior of each component process of a manageable part of the system. They then integrate these formulations to predict system behavior. In “bottom-up” PBPK modeling, the kinetic parameters extrapolated from both in vitro to in vivo using biologic scaling factors or those obtained by fitting can be used in the model construction [89]. This approach could be used to predict the kinetics of a new chemical entity based only on physiology and physicochemical properties. Mangold-Doring et al. developed a “bottom-up” multi-species PBTK model to predict steady-state bioconcentration factors (BCFs) for chemicals by Monte Carlo simulation, using model parameters derived from data on 69 freshwater fish species found in Canada. The predicted data were in general agreement with the empirical data [90]. In recent years, more and more researchers have mixed new data, known physiology, and pharmacological principles, as necessary, to construct a model. T’Jollyn et al. used the combination of “bottom-up” and “top-down” approaches, using a convenient probe substrate, which has the potential to update system-related parameters in order to better represent pediatric physiology [91].

### 6.3. Combination with Artificial Intelligence Techniques

With the rapid development of computer science, the artificial intelligence (AI) technique has provided significant approaches to improve the prediction of the ADME and other physiochemical properties. Machine learning (ML) methods, as a subset of AI, are combined with quantitative structure–activity relationship (QSAR) modeling to predict the key input parameters of the PBPK models. QSAR is a mathematical model approximating the often-complex relationships between chemical properties and biological activities of compounds [92]. Nowadays, QSAR has been successfully used in the model for predicting antiviral, antibacterial, and antidiabetic activities [93,94,95].

As early as 1998, Yang et al. developed this unconventional, efficient, and predictable toxicological method for toxicological assessment. Central to the approaches presented is the integration of physiologically based pharmacokinetic/pharmacodynamic (PBPK/PD) and quantitative structure–activity relationship (QSAR) modeling with focused mechanistically based experimental toxicology [96]. Chou established a PBPK model by integrating an ML-based quantitative QSAR model with a PBPK model to simulate the tumor-targeted delivery efficiency and biodistribution of various nanoparticles (NPs). This AI-PBPK model provides an efficient screening tool to rapidly predict the delivery efficiency of an NP based on its physicochemical properties without relying on an animal training data set [97]. Naga et al. minimized in vitro and in vivo testing in early drug discovery by combining PBPK and ML, and reduced the experimental cycle as much as possible. They also evaluated a new High-Throughput PBPK (HT-PBPK) method. Although the results of this method were similar to those of the conventional method, the simulation time was shortened from a few hours to a few seconds [98]. Integrating the predictive power of the PBPK model into ML can provide significant benefits in improving the accuracy and scope of drug screening and evaluation procedures [99].

In addition, AI has been applied to provide and optimize parameters required in model building. Bayesian models are a class of machine learning models that utilize Bayes’ theorem for computation, a combination of prior knowledge and new evidence to update probabilistic predictions. Combining the Bayesian model with the PBPK model can optimize the parameters of the PBPK model and make the model predictions more accurate [100]. Lin et al. used the Bayesian Markov Chain Monte Carlo (MCMC) algorithm to establish a PBPK model of tilapia to estimate the robustness of the stopping time of florfenicol under different physiological, environmental, and dosing conditions. The Bayesian–PBPK model provided new practical significance for improving food safety regulation [101]. Another study also utilized Bayesian correction to estimate the metabolic clearance and distribution coefficients of valproic acid and nine analogs in embryos, to better predict or infer the developmental toxicity of chemicals in zebrafish embryos [102].

## 7. Conclusions

In recent years, PBPK has increasingly been applied to study the behavior of inorganic pollutants, organic pollutants, and even some nanomaterials. For this reason, there was an urgent need to systematize the recent progress of PBPK. In this research, a total of 3974 PBPK-related articles were collected through the systematic literature review. The recent progress in medical and environmental research of the PBPK model was reviewed, and the major research directions in PBPK studies were also summarized by bibliometric method. Some general findings are as follows: (1) The bibliometric results showed that the number of PBPK-related articles has rapidly increased since 2014, which can be attributed to the development of computer technology. (2) Cross-species prediction, risk assessment, special population studies, and drug interactions are the four major directions in medical research. (3) Simulating the environmental behaviors and estimating the adverse effect are two major applications of PBPK in environmental research (4) The lack of parameters is the main problem affecting PBPK building and accuracy. The combination of quantitative structure–activity relationship, Bayesian method, and machine learning technology are potential solutions to the previous research gaps. It should be noted that our aim is not a simple comprehensive view of the applications of PBPK worldwide, but rather a summary of the progress in PBPK applications, revealing the major research fields in medical and environmental research, clarifying the gaps in previously provided PBPK studies, and finally offering insights into possible future areas of PBPK studies.

## Figures and Tables

**Table 1:** Top cited keywords in terms of frequency for the PBPK model.

No.	Keywords	Frequency	Centrality *
1	metabolism	391	0.37
2	risk assessment	345	0.65
3	drug–drug interaction	313	0.08
4	prediction	311	0.12
5	in vitro	309	0.09
6	tissue distribution	219	0.05
7	in vivo	211	0.03
8	disposition	200	0.06
9	exposure	168	0.16
10	clearance	131	0.13
11	absorption	124	0.02
12	simulation	115	0.01

* Centrality indicates the importance of the keyword in the co-occurrence map.

**Table 2:** PBPK application cases in four major research areas.

Model Types	Chemical	Model Object	Results	References
Multi-compartment model ^a^*	Spectinamide	Mice	A reduced PBPK model was developed to describe and predict the pharmacokinetics of spectinamide in various tissues	[35]
CD-PBPK model	Chloroprene	Rats Hamsters Humans	Development of IRIS evaluation of chloroprene	[36]
Multi-compartment model	Chlorpyrifos; 3,5,6-trichloro-2-pyridinol	Rats Adult humans	To predict urinary excretion of 3,5,6-trichloro-2-pyridinol (TCPY), the specific metabolite of chlorpyrifos (CPF), in young children	[37]
In vitro-to-in vivo extrapolation (IVIVE) approach for PBPK model	Carbaryl	Rats	A parameterized approach based on in vitro data was demonstrated to develop a physiologically based pharmacokinetic and pharmacodynamic (PBPK/PD) model that relates in vitro effective concentrations to human equivalent exposure	[9]
Multi-compartment model	Di-isobutyl phthalate; Mono-isobutyl phthalate	Rats	To develop and evaluate a physiologically based pharmacokinetic (PBPK) model for DiBP and MiBP in rats and extend this to human risk assessment based on human exposure	[38]
Multi-compartment model	TiO_2_	German	To simulate the biological distribution of nano TiO_2_. By calculating the daily dietary TiO_2_ intake of the population, this chronic external exposure was then translated into the internal titanium levels of each organ through the model	[39]
Multi-compartment model	Perfluorononanoic acid; Perfluorodecanoic acid	Rats	To detect PFNA and PFDA in male and female rats, and to apply the model to human health risk assessment for sex differences	[34]
Multi-compartment model	PFAAs	Monkeys	To develop a physiologically based pharmacokinetic (PBPK) model for PFOA and PFOS for monkeys and then scale this model to humans in order to describe available human drinking water data	[40]
Multi-compartment model	Patupilone (EPO906)	Rats	A novel PBPK model was developed based on rat tissue concentration data to predict blood concentration–time profiles of patupilone in cancer patients	[26]
Multi-compartment model	Atipamezole	Rats	To understand the underlying mechanisms of nonlinear PK in rats and linear PK in humans and develop physiologically based PK models (PBPK) to capture and validate this phenomenon	[41]
Multi-compartment model	AuNPs	Rats	To present the interspecies extrapolation of a PBK model initially developed for rats, in order to estimate the biodistribution of inhaled gold NPs (AuNPs) in humans	[32]
Whole-body PBPK model modeled from top to bottom (combination of multiple models)	Vicagrel; CYPs	Humans	To predict the in vivo drug–drug interaction (DDI) potential between vicagrel and bupropion as well as S-mephenytoin	[42]
Whole-body PBPK model modeled from top to bottom	Sonidegib; Ketoconazole; Rifampin	Healthy subjects and patients	Bridge the clinical drug–drug interaction (DDI) study of sonidegib with KTZ and RIF in healthy subjects to the marketed dos in patients; Predict acute versus long-term dosing of the perpetrators with sonidegib at a steady state; Predict the effect of moderate CYP3A perpetrators on sonidegib exposure in patients	[43]
Whole-body PBPK model	Cytochrome P450 enzymes (CYPs); Therapeutic protein (TPs); IL-6; Simvastatin	Patient	To quantitatively predict the impact of IL-6 on sensitive CYP3A4 substrates	[44]
Whole-body PBPK model modeled from top to bottom	Pravastatin	Humans	To predict the pharmacokinetics and drug–drug interactions (DDI) of pravastatin, using the in vitro transport parameters	[45]
PBPK models of four compounds were established respectively, and finally combined	S44121(Compounds used in cardiovascular diseases); Probenecid; Tenofovir; Ciprofloxacin	Monkeys; Humans	The results predicted by the model were compared with the results of clinical DDI studies and to investigate the interaction of S44121 with probenecid, tenofovir, and ciprofloxacin	[46]
Multi-compartment model	Nicotine Cotinine	Humans	The p-PBPK model reproduced the higher clearance rates of nicotine and cotinine in pregnant women than in non-pregnant women; Nicotine concentration reaches its maximum value within 2 min after an intravenous injection	[47]
Whole-body PBPK model	Oxycodone	Humans	The model successfully predicted the oxycodone disposition in adults, wherein the predicted versus observed AUC, Cmax, and Tmax were within 0.90 to 1.20-fold difference	[48]
Whole-body PBPK model	Caspofungin	Humans	There was no difference in the transport rate of OATP1B1 between CASLAMB and CASMTD patients in the PBPK model; The model was able to sufficiently predict the pharmacokinetics of pediatric patients compared to published data	[49]
Whole-body PBPK model	Infliximab	Humans	To assess the pharmacokinetic and different speed reductions in infliximab (Remicade), and will be single resistance to pharmacokinetic knowledge from the accuracy of the adult is passed to the child	[50]
Whole-body PBPK model	Buprenorphine	Humans	To predict the pharmacokinetics of buprenorphine in pediatrics using the PBPK model	[51]
Whole-body PBPK model	Clindamycin	Humans	Used the pediatric PBPK model to optimize intravenous clindamycin dosing for a future prospective validation trial	[52]

^a^* The blood is the central medium, and the rest of the body’s organs and tissues are major subdivisions.

**Table 3:** Research on PBPK model for environmental research.

Model Types	Pollutant	Species	Results	Conclusion	References
Multi-compartment model ^a^*	Cu Zn	Human	Investigated the respiratory exposure characteristics and health risks of Cu and Zn from particles with PM_2.5_ in five microenvironments by using a chronic non-carcinogenic risk index and a physiologically based pharmacokinetic (PBPK) model	The results of exposure assessment based on the PBPK model indicated that the concentrations of Cu and Zn from PM_2.5_ were high in the liver and kidney but low in arterial and venous blood. After respiratory exposure was stopped and the pollutant concentrations reached a steady state, the highest concentrations of Cu and Zn were found in muscles. The muscles and brain exhibited the highest internal exposure risk index values for Cu and Zn	[81]
Multi-compartment model	As (V)	Medaka	Biotransport (uptake, distribution, and elimination) and biotransformation of As (V) in Marine medaka after exposure to water were simulated using radiotracer techniques and a PBPK model	The gut and gills are the sites of arsenic absorption, exchange, and elimination, and the body and head are the main storage sites	[79]
Multi-compartment model	Bisphenol A 4-nonylphenol Triclosan	Nile tilapia	Improving the performance of conventional PBTK models by modeling the PBTK-bound metabolism of bisphenol a, 4-nonylphenol, and Triclosan (PBTK-MT)	PBTK-MT showed high accuracy in predicting the concentrations of the three compounds in fish. This model contributes to a better understanding of the environmental behavior and risk of contaminants in aquatic populations	[82]
PBPK-CFD-CSP ^b^* hybrid analysis model	Formaldehyde	computer simulated person	Employed a newly developed CSP, which integrated the actual shape of the human body geometry with a virtual airway reproduced realistic human respiratory tract. In addition, PBPK-CFD hybrid analysis was integrated into the CSP-based numerical simulation to estimate inhalation exposure and respiratory tissue dosimetry with the unsteady breathing cycle model	Heterogeneous and transient contaminant concentration in indoor spaces, and time-dependent inhaled formaldehyde concentration including adsorption distributions, i.e., heterogeneous tissue dosimetry in the respiratory tract were precisely analyzed	[83]
Multi-compartment model	OCPs; PAHs; PFOS; DDT; Pyr	62 species of wild fish	The physiological parameters of fish were used to optimize the PBPK model to obtain the effective concentration (EC) thresholds, which could be used to assess the risks of POPs to fish in remote areas	Tibetan *Schizothorax* and *Macroschizothorax* are the most vulnerable Tibetan species; The risk of newly emerged POPs (PFOS) was 2–3 orders of magnitude higher than that of legacy POPs (DDT and pyridine)	[84]

^a^* The blood is the central medium, and the rest of the body’s organs and tissues are major subdivisions. ^b^* computational fluid dynamic (CFD), computer-simulated person (CSP).

## Data Availability

Data are contained within the article.

## Supplementary Materials

The following supporting information can be downloaded at: https://www.mdpi.com/article/10.3390/toxics12060433/s1, Figure S1: Co-occurrence network of keywords in the drug assessment research field; Figure S2: Co-occurrence network of keywords in the cross-species prediction research field; Figure S3: Co-occurrence network of keywords in the drug–drug interactions research field; Figure S4: Co-occurrence network of keywords in the pediatrics and pregnancy drug development research field.

## References

[1] Kuepfer L., Niederalt C., Wendl T., Schlender J.F., Willmann S., Lippert J., Block M., Eissing T., Teutonico D.J. (2016). Applied concepts in PBPK modeling: How to build a PBPK/PD model. CPT Pharmacomet. Syst. Pharmacol..

[2] Nestorov I.J. (2003). Whole body pharmacokinetic models. Clin. Pharmacokinet..

[3] Krstevska A., Duris J., Ibric S., Cvijic S. (2023). In-Depth Analysis of Physiologically Based Pharmacokinetic (PBPK) Modeling Utilization in Different Application Fields Using Text Mining Tools. Pharmaceutics.

[4] Teorell T.J. (1937). Kinetics of distribution of substances administered to the body, I: The extravascular modes of administration. Arch. Int. Pharmacodyn. Ther..

[5] Bellman R., Jacquez J.A., Kalaba R.J. (1960). Some mathematical aspects of chemotherapy: I. One-organ models. Bull. Math. Biophys..

[6] Dedrick R.L. (1973). Animal scale-up. J. Pharmacokinet. Biopharm..

[7] Nestorov I.J. (2007). Whole-body physiologically based pharmacokinetic models. Expert Opin. Drug Metab. Toxicol..

[8] Yang F., Sun N., Sun Y.X., Shan Q., Zhao H.Y., Zeng D.P., Zeng Z.L. (2013). A physiologically based pharmacokinetics model for florfenicol in crucian carp and oral-to-intramuscular extrapolation. J. Vet. Pharmacol. Ther..

[9] Yoon M., Kedderis G.L., Yan G.Z., Clewell H.J. (2015). Use of in vitro data in developing a physiologically based pharmacokinetic model: Carbaryl as a case study. Toxicology.

[10] Simmons J.E., Evans M.V., Boyes W.K. (2005). Moving from external exposure concentration to internal dose: Duration extrapolation based on physiologically based pharmacokinetic derived estimates of internal dose. J. Toxicol. Environ. Health-Part A-Curr. Issues.

[11] Björkman S., Wada D.R., Berling B.M., Benoni G.J. (2001). Prediction of the disposition of midazolam in surgical patients by a physiologically based pharmacokinetic model. J. Pharm. Sci..

[12] Frederick C.B., Lomax L.G., Black K.A., Finch L., Scribner H.E., Kimbell J.S., Morgan K.T., Subramaniam R.P., Morris J.B. (2002). Use of a hybrid computational fluid dynamics and physiologically based inhalation model for interspecies dosimetry comparisons of ester vapors. Toxicol. Appl. Pharmacol..

[13] Hosea N.A., Collard W.T., Cole S., Maurer T.S., Fang R.X., Jones H., Kakar S.M., Nakai Y., Smith B.J., Webster R. (2009). Prediction of Human Pharmacokinetics from Preclinical Information: Comparative Accuracy of Quantitative Prediction Approaches. J. Clin. Pharmacol..

[14] Mahmood I.J. (1998). Interspecies scaling of renally secreted drugs. Life Sci..

[15] Jones H.M., Parrott N., Jorga K., Lavé T.J. (2006). A novel strategy for physiologically based predictions of human pharmacokinetics. Clin. Pharmacokinet..

[16] Gallo J.M., Vicini P., Orlansky A., Li S., Zhou F., Ma J., Pulfer S., Bookman M.A., Guo P.J. (2004). Pharmacokinetic model-predicted anticancer drug concentrations in human tumors. Clin. Cancer Res..

[17] Liu X., Smith B.J., Chen C., Callegari E., Becker S.L., Chen X., Cianfrogna J., Doran A.C., Doran S.D., Gibbs J.P. (2005). Use of a physiologically based pharmacokinetic model to study the time to reach brain equilibrium: An experimental analysis of the role of blood-brain barrier permeability, plasma protein binding, and brain tissue binding. J. Pharmacol. Exp. Ther..

[18] Clewell R.A., Merrill E.A., Yu K.O., Mahle D.A., Sterner T.R., Fisher J.W., Gearhart J.M. (2003). Predicting neonatal perchlorate dose and inhibition of iodide uptake in the rat during lactation using physiologically-based pharmacokinetic modeling. Toxicol. Sci..

[19] Corley R.A., Mast T.J., Carney E.W., Rogers J.M., Daston G.P. (2003). Evaluation of physiologically based models of pregnancy and lactation for their application in children’s health risk assessments. Crit. Rev. Toxicol..

[20] Biesdorf C., Martins F.S., Sy S.K., Diniz A.J. (2019). Physiologically-based pharmacokinetics of ziprasidone in pregnant women. Br. J. Clin. Pharmacol..

[21] Villesen H.H., Foster D.J., Upton R.N., Somogyi A.A., Martinez A., Grant C.J. (2006). Cerebral kinetics of oxycodone in conscious sheep. J. Pharm. Sci..

[22] Knobloch M., Portier C., Levionnois O., Theurillat R., Thormann W., Spadavecchia C., Mevissen M.J. (2006). Antinociceptive effects, metabolism and disposition of ketamine in ponies under target-controlled drug infusion. Toxicol. Appl. Pharmacol..

[23] Zhou Y., Zhuoga S., Chen Y., Wang X., Fu J., Zhou W., Gao S. (2023). Optimizing the physiological pharmacokinetic model to rank the risks of persistent organic pollutants towards fish on the Tibetan Plateau. Sci. Total Environ..

[24] Wintermyer M., Skaidas A., Roy A., Yang Y.C., Georgapoulos P., Burger J., Cooper K. (2005). The development of a physiologically-based pharmacokinetic model using the distribution of 2 317 8-tetrachlorodibenzo-p-dioxin in the tissues of the eastern oyster (*Crassostrea virginica*). Mar. Environ. Res..

[25] Chen S., Xu Y. (2011). Methods for evaluating the bioavailability of organic contaminants in environments. Environ. Chem..

[26] Sager J., Yu J., Ragueneau-Majlessi I., Isoherranen N. (2015). Physiologically based pharmacokinetic (PBPK) modeling and simulation approaches: A systematic review of published models, applications, and model verification. Drug Metab. Dispos..

[27] Bustamante P., Bocher P., Chérel Y., Miramand P., Caurant F. (2003). Distribution of trace elements in the tissues of benthic and pelagic fish from the Kerguelen Islands. Sci. Total Environ..

[28] Chen Y., Mao J., Hop C.E. (2015). Physiologically based pharmacokinetic modeling to predict drug-drug interactions involving inhibitory metabolite: A case study of amiodarone. Drug Metab. Dispos..

[29] Verner M.-A., Ayotte P., Muckle G., Charbonneau M., Haddad S. (2009). A Physiologically Based Pharmacokinetic Model for the Assessment of Infant Exposure to Persistent Organic Pollutants in Epidemiologic Studies. Environ. Health Perspect..

[30] Andersen M., Clewell III H., Gargas M., Smith F., Reitz R.J. (1987). Physiologically based pharmacokinetics and the risk assessment process for methylene chloride. Toxicol. Appl. Pharmacol..

[31] Xia B., Heimbach T., Lin T.-h., He H., Wang Y., Tan E.J. (2012). Novel physiologically based pharmacokinetic modeling of patupilone for human pharmacokinetic predictions. Cancer Chemother. Pharmacol..

[32] Gakis G., Krikas A., Neofytou P., Tran L., Charitidis C.J. (2022). Modelling the biodistribution of inhaled gold nanoparticles in rats with interspecies extrapolation to humans. Toxicol. Appl. Pharmacol..

[33] Yang M., Wang A.Q., Padilha E.C., Shah P., Hagen N.R., Shamim K., Huang W., Xu X.J. (2023). Use of physiological based pharmacokinetic modeling for cross-species prediction of pharmacokinetic and tissue distribution profiles of a novel niclosamide prodrug. Front. Pharmacol..

[34] Kim S.J., Choi E.J., Choi G.W., Lee Y.B., Cho H.Y. (2019). Exploring sex differences in human health risk assessment for PFNA and PFDA using a PBPK model. Arch. Toxicol..

[35] Parmar K.R., Lukka P.B., Wagh S., Temrikar Z.H., Liu J., Lee R.E., Braunstein M., Hickey A.J., Robertson G.T., Gonzalez-Juarrero M. (2023). Development of a Minimalistic Physiologically Based Pharmacokinetic (mPBPK) Model for the Preclinical Development of Spectinamide Antibiotics. Pharmaceutics.

[36] DeWoskin R.S. (2007). PBPK models in risk assessment—A focus on chloroprene. Chem. Biol. Interact..

[37] Lu C., Holbrook C.M., Andres L.M. (2010). The Implications of Using a Physiologically Based Pharmacokinetic (PBPK) Model for Pesticide Risk Assessment. Environ. Health Perspect..

[38] Jeong S.-H., Jang J.-H., Cho H.-Y., Lee Y.B. (2021). Human risk assessment of di-isobutyl phthalate through the application of a developed physiologically based pharmacokinetic model of di-isobutyl phthalate and its major metabolite mono-isobutyl phthalate. Arch. Toxicol..

[39] Bachler G., von Goetz N., Hungerbuhler K.J. (2015). Using physiologically based pharmacokinetic (PBPK) modeling for dietary risk assessment of titanium dioxide (TiO2) nanoparticles. Nanotoxicology.

[40] Loccisano A.E., Campbell J.L., Andersen M.E., Clewell H.J. (2011). Evaluation and prediction of pharmacokinetics of PFOA and PFOS in the monkey and human using a PBPK model. Regul. Toxicol. Pharmacol..

[41] Li Z., Gao Y., Yang C., Xiang Y., Zhang W., Zhang T., Su R., Lu C., Zhuang X.J. (2020). Assessment and confirmation of species difference in nonlinear pharmacokinetics of atipamezole with physiologically based pharmacokinetic modeling. Drug Metab. Dispos..

[42] Liu S., Wang Z., Chan E., Zhao Y., Kang J., Zhang X., Tian X.J. (2022). Inhibition of cytochrome P450 enzymes and uridine 5′-diphospho-glucuronosyltransferases by vicagrel in human liver microsomes: A prediction of potential drug-drug interactions. Chem. -Biol. Interact..

[43] Einolf H.J., Zhou J., Won C., Wang L., Rebello S.J. (2017). A physiologically-based pharmacokinetic modeling approach to predict drug–drug interactions of sonidegib (LDE225) with perpetrators of CYP3A in cancer patients. Drug Metab. Dispos..

[44] Machavaram K., Almond L., Rostami-Hodjegan A., Gardner I., Jamei M., Tay S., Wong S., Joshi A., Kenny J.J. (2013). A physiologically based pharmacokinetic modeling approach to predict disease–drug interactions: Suppression of CYP3A by IL-6. Clin. Pharmacol. Ther..

[45] Varma M.V., Lai Y., Feng B., Litchfield J., Goosen T.C., Bergman A.J. (2012). Physiologically based modeling of pravastatin transporter-mediated hepatobiliary disposition and drug-drug interactions. Pharm. Res..

[46] Ball K., Jamier T., Parmentier Y., Denizot C., Mallier A., Chenel M.J. (2017). Prediction of renal transporter-mediated drug-drug interactions for a drug which is an OAT substrate and inhibitor using PBPK modelling. Eur. J. Pharm. Sci..

[47] Amice B., Ho H., Zhang E., Bullen C.J. (2021). Physiologically based pharmacokinetic modelling for nicotine and cotinine clearance in pregnant women. Front. Pharmacol..

[48] Zheng L., Xu M., Tang S.-W., Song H.-X., Jiang X.-H., Wang L. (2019). Physiologically Based Pharmacokinetic Modeling of Oxycodone in Children to Support Pediatric Dosing Optimization. Pharm. Res..

[49] Stader F., Wuerthwein G., Groll A.H., Vehreschild J.-J., Cornely O.A., Hempel G. (2015). Physiology-Based Pharmacokinetics of Caspofungin for Adults and Paediatrics. Pharm. Res..

[50] Malik P.R., Edginton A.N. (2019). Physiologically-Based Pharmacokinetic Modeling vs. Allometric Scaling for the Prediction of Infliximab Pharmacokinetics in Pediatric Patients. Cpt-Pharmacomet. Syst. Pharmacol..

[51] Kovar L., Schraepel C., Selzer D., Kohl Y., Bals R., Schwab M., Lehr T. (2020). Physiologically-Based Pharmacokinetic (PBPK) Modeling of Buprenorphine in Adults, Children and Preterm Neonates. Pharmaceutics.

[52] Hornik C.P., Wu H., Edginton A.N., Watt K., Cohen-Wolkowiez M., Gonzalez D. (2017). Development of a Pediatric Physiologically-based Pharmacokinetic Model of Clindamycin Using Opportunistic Pharmacokinetic Data. Clin. Pharmacokinet..

[53] Upton R.N., Foster D.J., Abuhelwa A.Y. (2016). An introduction to physiologically-based pharmacokinetic models. Pediatr. Anesth..

[54] Brinkmann M., Schlechtriem C., Reininghaus M., Eichbaum K., Buchinger S., Reifferscheid G., Hollert H., Preuss T.G. (2016). Cross-Species Extrapolation of Uptake and Disposition of Neutral Organic Chemicals in Fish Using a Multispecies Physiologically-Based Toxicokinetic Model Framework. Environ. Sci. Technol..

[55] Gingrich J., Filipovic D., Conolly R., Bhattacharya S., Veiga-Lopez A. (2021). Pregnancy-specific physiologically-based toxicokinetic models for bisphenol A and bisphenol S. Environ. Int..

[56] Komiya H., Umegaki H., Asai A., Kanda S., Maeda K., Shimojima T., Nomura H., Kuzuya M. (2018). Factors associated with polypharmacy in elderly home-care patients. Geriatr. Gerontol. Int..

[57] Varma M.V., Pang K.S., Isoherranen N., Zhao P.J. (2015). Dealing with the complex drug–drug interactions: Towards mechanistic models. Biopharm. Drug Dispos..

[58] Zhang L., Zhang Y., Zhao P., Huang S.-M. (2009). Predicting Drug-Drug Interactions: An FDA Perspective. Aaps J..

[59] Posada M.M., Bacon J.A., Schneck K.B., Tirona R.G., Kim R.B., Higgins J.W., Pak Y.A., Hall S.D., Hillgren K.M. (2015). Prediction of renal transporter mediated drug-drug interactions for pemetrexed using physiologically based pharmacokinetic modeling. Drug Metab. Dispos..

[60] Sychterz C., Gardner I., Chiang M., Rachumallu R., Neuhoff S., Perera V., Merali S., Schmidt B.J., Gaohua L.J. (2022). Performance verification of CYP2C19 enzyme abundance polymorphism settings within the simcyp simulator v21. Metabolites.

[61] Yamashita F., Sasa Y., Yoshida S., Hisaka A., Asai Y., Kitano H., Hashida M., Suzuki H.J. (2013). Modeling of rifampicin-induced CYP3A4 activation dynamics for the prediction of clinical drug-drug interactions from in vitro data. PLoS ONE.

[62] Cheong E.J., Goh J.J., Hong Y., Kojodjojo P., Chan E.C. (2018). Rivaroxaban with and without amiodarone in renal impairment. J. Am. Coll. Cardiol..

[63] Einolf H.J. (2007). Comparison of different approaches to predict metabolic drug-drug interactions. Xenobiotica.

[64] Fahmi O.A., Boldt S., Kish M., Obach R.S., Tremaine L.M. (2008). Prediction of drug-drug interactions from in vitro induction data: Application of the relative induction score approach using cryopreserved human hepatocytes. Drug Metab. Dispos..

[65] Fowler S., Morcos P.N., Cleary Y., Martin-Facklam M., Parrott N., Gertz M., Yu L.J. (2017). Progress in prediction and interpretation of clinically relevant metabolic drug-drug interactions: A minireview illustrating recent developments and current opportunities. Curr. Pharmacol. Rep..

[66] Jones H., Chen Y., Gibson C., Heimbach T., Parrott N., Peters S., Snoeys J., Upreti V., Zheng M., Hall S.J. (2015). Physiologically based pharmacokinetic modeling in drug discovery and development: A pharmaceutical industry perspective. Clin. Pharmacol. Ther..

[67] Nordmark A., Andersson A., Baranczewski P., Wanag E., Ståhle L.J. (2014). Assessment of interaction potential of AZD2066 using in vitro metabolism tools, physiologically based pharmacokinetic modelling and in vivo cocktail data. Eur. J. Clin. Pharmacol..

[68] Duan P., Fisher J.W., Yoshida K., Zhang L., Burckart G.J., Wang J. (2017). Physiologically Based Pharmacokinetic Prediction of Linezolid and Emtricitabine in Neonates and Infants. Clin. Pharmacokinet..

[69] Gentry P.R., Covington T.R., Clewell H.J. (2003). Evaluation of the potential impact of pharmacokinetic differences on tissue dosimetry in offspring during pregnancy and lactation. Regul. Toxicol. Pharmacol..

[70] Pelekis M., Gephart L.A., Lerman S.E. (2001). Physiological-model-based derivation of the adult and child pharmacokinetic intraspecies uncertainty factors for volatile organic compounds. Regul. Toxicol. Pharmacol..

[71] Price K., Haddad S., Krishnan K. (2003). Physiological modeling of age-specific changes in the pharmacokinetics of organic chemicals in children. J. Toxicol. Environ. Health. Part A.

[72] Ginsberg G., Hattis D., Russ A., Sonawane B. (2004). Physiologically based pharmacokinetic (PBPK) modeling of caffeine and theophylline in neonates and adults: Implications for assessing children’s risks from environmental agents. J. Toxicol. Environ. Health-Part A-Curr. Issues.

[73] Mackay D., Fraser A.J. (2000). Bioaccumulation of persistent organic chemicals: Mechanisms and models. Environ. Pollut..

[74] Clewell H.J., Crump K.S., Gentry P.R., Shipp A.M. (2000). Site-specific reference dose for methylmercury for fish-eating populations. Fuel Process. Technol..

[75] Wang W.-X., Tan Q.G. (2019). Applications of dynamic models in predicting the bioaccumulation, transport and toxicity of trace metals in aquatic organisms. Environ. Pollut..

[76] Weijs L., Covaci A., Yang R.S., Das K., Blust R. (2011). A non-invasive approach to study lifetime exposure and bioaccumulation of PCBs in protected marine mammals: PBPK modeling in harbor porpoises. Toxicol. Appl. Pharmacol..

[77] Weijs L., Covaci A., Yang R.S., Das K., Blust R. (2012). Computational toxicology: Physiologically based pharmacokinetic models (PBPK) for lifetime exposure and bioaccumulation of polybrominated diphenyl ethers (PBDEs) in marine mammals. Environ. Pollut..

[78] Taylor A.C., Fones G.R., Vrana B., Mills G.A. (2021). Applications for passive sampling of hydrophobic organic contaminants in water—A review. Crit. Rev. Anal. Chem..

[79] Zhang W., Song D.D., Tan Q.G., Wang W.X., Zhang L. (2020). Physiologically Based Pharmacokinetic Model for the Biotransportation of Arsenic in Marine Medaka (*Oryzias melastigma*). Environ. Sci. Technol..

[80] Stadnicka J., Schirmer K., Ashauer R. (2012). Predicting concentrations of organic chemicals in fish by using toxicokinetic models. Environ. Sci. Technol..

[81] Li Y.N., Wang B.Q., Wu J.C., Xu X.F., Guo A., Ji Y.Q. (2023). Risk of chronic non-carcinogenic and internal respiratory exposure to Cu and Zn from particulate matter 2.5 in different microenvironments. Urban Clim..

[82] Liu Y.-H., Yao L., Huang Z., Zhang Y.-Y., Chen C.-E., Zhao J.-L., Ying G.-G. (2022). Enhanced prediction of internal concentrations of phenolic endocrine disrupting chemicals and their metabolites in fish by a physiologically based toxicokinetic incorporating metabolism (PBTK-MT) model. Environ. Pollut..

[83] Yoo S.J., Ito K. (2018). Assessment of transient inhalation exposure using human model integrated with PBPK-CFD hybrid analysis. Sustain. Cities Soc..

[84] Zhang S., Wang Z., Chen J.J. (2019). Physiologically based toxicokinetics (PBTK) models for pharmaceuticals and personal care products in wild common carp (*Cyprinus carpio*). Chemosphere.

[85] Sonne C., Gustayson K., Riget F.F., Dietz R., Kruger T., Bonefeld-Jorgensen E.C. (2014). Physiologically based pharmacokinetic modeling of POPs in Greenlanders. Environ. Int..

[86] Ling M.-P., Wu C.-H., Chen S.-C., Chen W.-Y., Chio C.-P., Cheng Y.-H., Liao C.-M. (2014). Probabilistic framework for assessing the arsenic exposure risk from cooked fish consumption. Environ. Geochem. Health.

[87] Liao C.M., Liang H.M., Chen B.C., Singh S., Tsai J.W., Chou Y.H., Lin W.T. (2005). Dynamical coupling of PBPK/PD and AUC-based toxicity models for arsenic in tilapia Oreochromis mossambicus from blackfoot disease area in Taiwan. Environ. Pollut.

[88] Rescigno A. (2010). The Two Faces of Pharmacokinetics. J. Pharm. Pharm. Sci..

[89] Sato M., Toshimoto K., Tomaru A., Yoshikado T., Tanaka Y., Hisaka A., Lee W., Sugiyama Y. (2018). Physiologically Based Pharmacokinetic Modeling of Bosentan Identifies the Saturable Hepatic Uptake as a Major Contributor to Its Nonlinear Pharmacokinetics. Drug Metab. Dispos..

[90] Mangold-Doring A., Grimard C., Green D., Petersen S., Nichols J.W., Hogan N., Weber L., Hollert H., Hecker M., Brinkmann M. (2021). A Novel Multispecies Toxicokinetic Modeling Approach in Support of Chemical Risk Assessment. Environ. Sci. Technol..

[91] T’Jollyn H., Vermeulen A., Van Bocxlaer J. (2019). PBPK and its Virtual Populations: The Impact of Physiology on Pediatric Pharmacokinetic Predictions of Tramadol. Aaps J..

[92] Eriksson L., Jaworska J., Worth A.P., Cronin M.T., McDowell R.M., Gramatica P. (2003). Methods for reliability and uncertainty assessment and for applicability evaluations of classification- and regression-based QSARs. Environ. Health Perspect..

[93] Sabadini G., Mellado M., Morales C., Mella J. (2024). Arylamines QSAR-Based design and molecular dynamics of new phenylthiophene and benzimidazole derivatives with affinity for the C111, Y268, and H73 Sites of SARS-CoV-2 PLpro enzyme. Pharmaceuticals.

[94] Knaak J., Dary C., Zhang X., Gerlach R., Tornero-Velez R., Chang D., Goldsmith R., Blancato J. (2012). Parameters for pyrethroid insecticide QSAR and PBPK/PD models for human risk assessment. Rev. Environ. Contam. Toxicol..

[95] Deepika D., Kumar V. (2023). The role of “physiologically based pharmacokinetic model (PBPK)” new approach methodology (NAM) in pharmaceuticals and environmental chemical risk assessment. Int. J. Environ. Res. Public Health.

[96] Yang R., Thomas R., Gustafson D., Campain J., Benjamin S., Verhaar H., Mumtaz M. (1998). Approaches to developing alternative and predictive toxicology based on PBPK/PD and QSAR modeling. Environ. Health Perspect..

[97] Chou W.C., Cheng Y.H., Riviere J.E., Monteiro-Riviere N.A., Kreyling W.G., Lin Z.M. (2022). Development of a multi-route physiologically based pharmacokinetic (PBPK) model for nanomaterials: A comparison between a traditional versus a new route-specific approach using gold nanoparticles in rats. Part. Fibre Toxicol..

[98] Naga D., Parrott N., Ecker G.F., Olivares-Morales A. (2022). Evaluation of the Success of High-Throughput Physiologically Based Pharmacokinetic (HT-PBPK) Modeling Predictions to Inform Early Drug Discovery. Mol. Pharm..

[99] Habiballah S., Reisfeld B. (2023). Adapting physiologically-based pharmacokinetic models for machine learning applications. Sci. Rep..

[100] Bernillon P., Bois F.Y. (2000). Statistical issues in toxicokinetic modeling: A Bayesian perspective. Environ. Health Perspect..

[101] Lin H.-C., Chen W.-Y. (2021). Bayesian population physiologically-based pharmacokinetic model for robustness evaluation of withdrawal time in tilapia aquaculture administrated to florfenicol. Ecotoxicol. Environ. Saf..

[102] Simeon S., Brotzmann K., Fisher C., Gardner I., Silvester S., Maclennan R., Walker P., Braunbeck T., Bois F.Y. (2020). Development of a generic zebrafish embryo PBPK model and application to the developmental toxicity assessment of valproic acid analogs. Reprod. Toxicol..

