# Supplementary material for: Recent Progress on Physiologically Based Pharmacokinetic (PBPK) Model: A Review Based on Bibliometrics
Source: Toxics. 2024 Jun 14;12(6):433. doi: 10.3390/toxics12060433 (PMC11209072; doi:10.3390/toxics12060433)
Supplement: Supplementary file 1 [file toxics-12-00433-s001.zip › Supplementary Material.pdf]

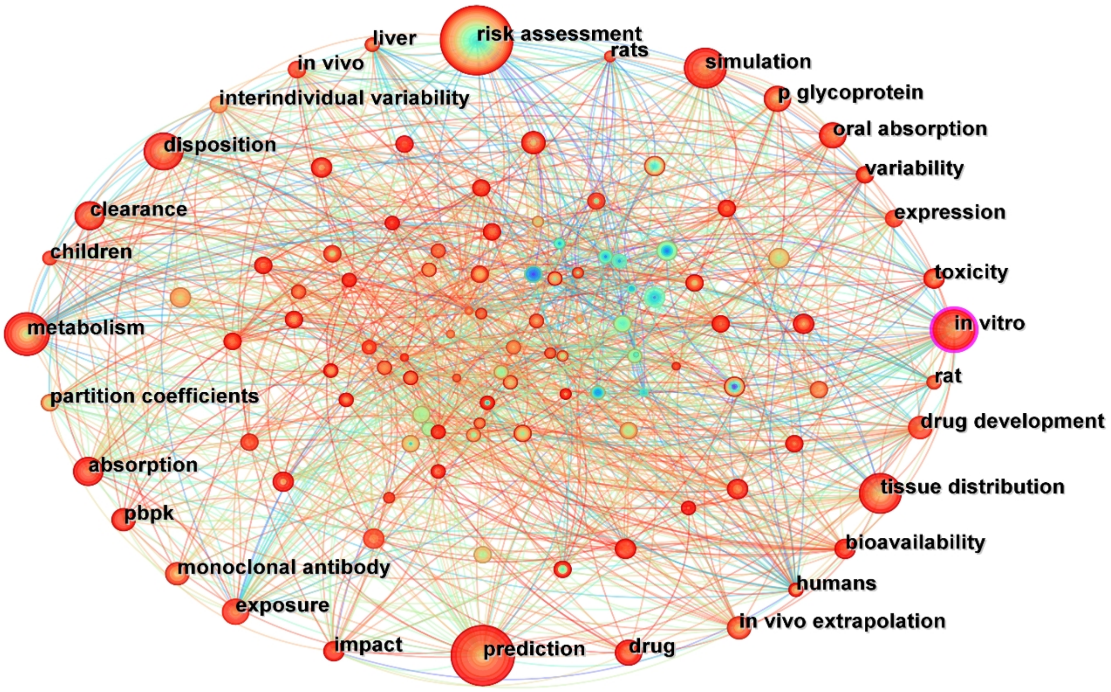

Figure S1. Co-occurrence network of keywords in the drug assessment research field

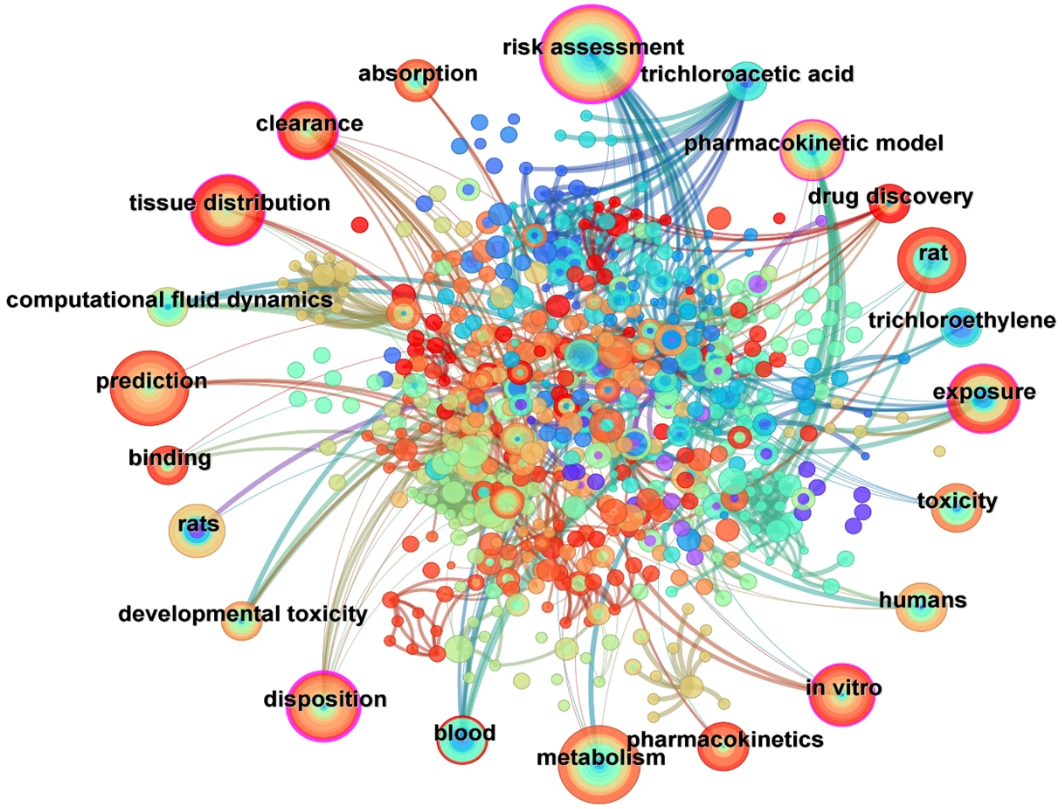

Figure S2. Co-occurrence network of keywords in the cross species prediction research field

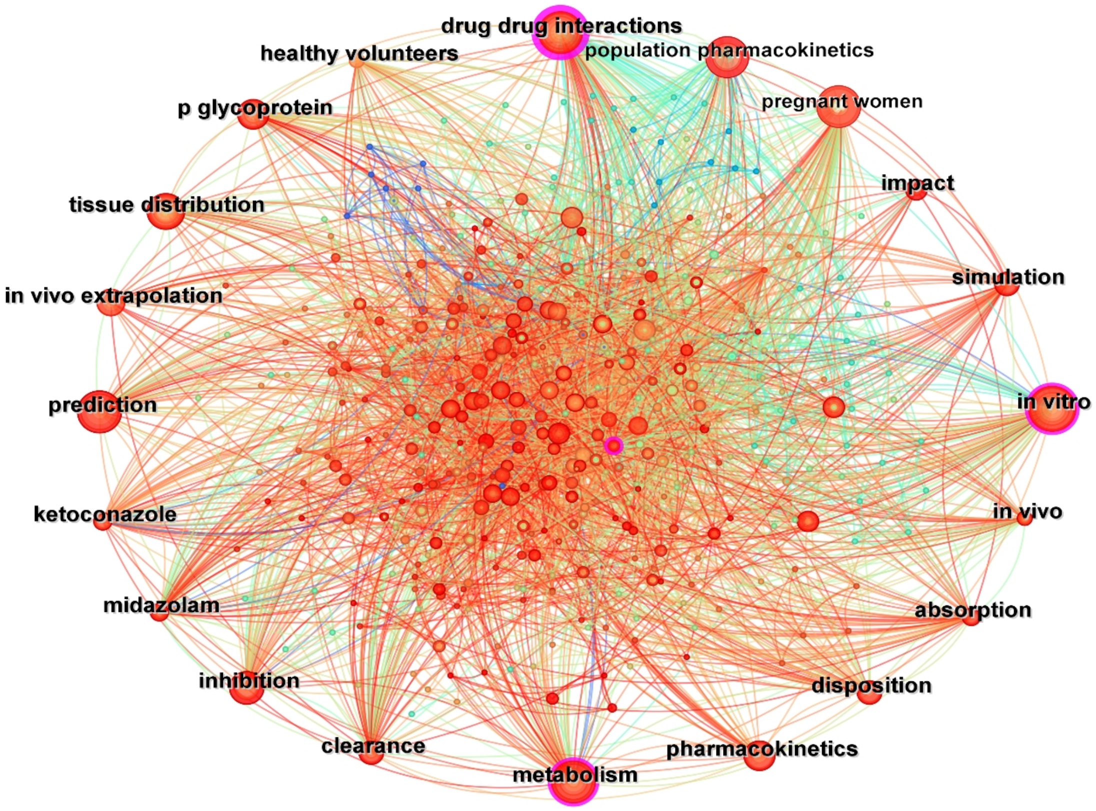

Figure S3. Co-occurrence network of keywords in the drug-drug interactions research field

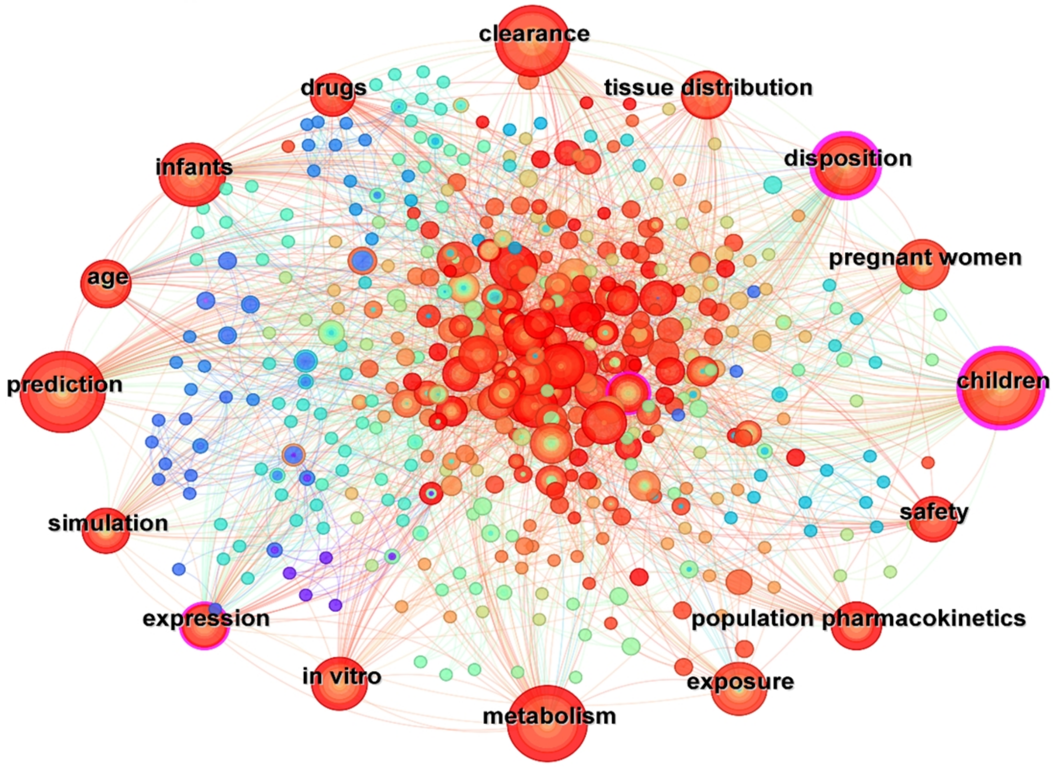

Figure S4. Co-occurrence network of keywords in the pediatrics and pregnancy drug development research field
